# Developing a Virtual Multicultural Intervention for University Students

**DOI:** 10.3390/bs10110168

**Published:** 2020-11-05

**Authors:** Kristen Black, Manyu Li

**Affiliations:** Department of Psychology, University of Louisiana at Lafayette, P. O. Box 43644, Lafayette, LA 70504, USA; kmblck22@gmail.com

**Keywords:** multiculturalism, intergroup relations, intervention, cultural sensitivity, diversity awareness

## Abstract

The recent surge in acts of violence motivated by intergroup biases in the United States are of great concern. If allowed to progress, these conditions could create an unwelcoming atmosphere and could foster further division within the United States. Based on previous culture-related studies, multiculturalism is a possible solution to reducing intergroup biases, as it positively affects implicit and explicit cultural attitudes, perceptions, as well as behaviors. The current study developed a virtual multiculturalism intervention as a means to improve intergroup relations and combat cultural biases within the undergraduate student population. Specifically, 249 undergraduate students were randomly assigned to the intervention condition and the control condition. Results showed that participants in the intervention condition had a significantly higher improvement in multiculturalism scores, supporting the incorporation of a virtual multiculturalism intervention in higher education. This study calls for the implementation of a better framework of understanding of how changes in multicultural events are perceived and how this can be used to create a more empathetic population who are more comfortable and understanding with one another. This inexpensive and timesaving model holds the possibility of being used in the future to aid in overcoming cultural differences between student populations and varying ethnics groups alike.

“*We all should know that diversity makes for a rich tapestry, and we must understand that all the threads of the tapestry are equal in value no matter what their color.*”

—Maya Angelou

## 1. Introduction

This study aims at developing a virtual multicultural intervention for college students to reduce intergroup conflicts on race/ethnicity issues and to increase multiculturalism. In 2017, within the United States (US), a total of 7175 hate crimes were reported to the Federal Bureau of Investigation. About 58% of single-bias hate incidents recorded were racially motivated [1]. According to The Sentencing Project [2], African-Americans and Latinos make up 29% of the US population but comprise 57% of the US prison population. Other racial disparities can be found in a study observing police–civilian interactions via body cameras. The results showed that during routine traffic stops, officers were seen to speak significantly less respectfully to Black community members compared to their Caucasian counterparts [3]. Racially motivated acts of prejudice are widespread, making up 33.9% of all discrimination charges [4]. Another form of cultural bias that is not reflected in criminal data is cultural appropriation, which is defined in the literature as the adoption and misuse of symbols, artifacts, genres, rituals, or technologies associated with a particular culture or subculture [5].

As the US continues to grow in population, its racial profile and overall diversity are expected to grow. The US is projected to become a plurality of racial and ethnic groups—with non-Hispanic Caucasians no longer being a majority [6]. In order to prepare individuals for a more global, pluralistic society, multiculturalism should be incorporated into the teaching curriculum. Implementing multiculturalism is pertinent “to reduce the misunderstandings across subcultures” and to foster a sense of respect for those with other identities unlike our own [7] (p. 29). The multicultural intervention developed in this study addressed issues such as hate crime and also implicit biases, such as cultural appropriation. 

### 1.1. Definition of Multiculturalism

Multiculturalism can be defined as “a social-intellectual movement that promotes the value of diversity as a core principle and insists that all cultural groups be treated with respect and as equals” [8] (p. 609). In this definition of multiculturalism, cultural groups are not limited to ethnicity and race. According to the North American Psychological Association’s multicultural guidelines, group identities also include disability, age, gender, religion/spirituality, sexual orientation, and social class [9]. In this study, intervention methods were used to expose participants to multiculturalism educational materials. Intervention studies are programs that draw attention to specific behaviors or attitudes and then build up other behaviors or attitudes. Although the intervention developed in this study mostly concerns racial/ethnic groups, the intervention materials will also briefly cover other group identities.

### 1.2. Multiculturalism Intervention

Cultural groups are not limited to ethnicity and race. This study uses a more multicultural approach when employing intervention studies. Multicultural intervention studies are intervention studies that draw primary focus to concerns regarding racial and ethical issues. Previous studies have analyzed the effects of multiculturalism interventions on various populations, ranging from primary education to social work professionals [10]. These studies all displayed the positive effects of teaching multiculturalism. For example, Smith’s [10] two-group intervention study analyzed the impact of “culture school” on registered nurses’ levels of cultural competency. The culture school was based on a transcultural assessment theoretical model that emphasizes cultural awareness, acknowledging personal biases, and recognizing diversity issues. Subjects’ cultural knowledge and cultural self-efficacy were assessed pre-intervention and post-intervention, as well as 3 weeks after the study. The findings of this experiment suggested that educational, multiculturalism-based interventions can significantly increase cultural competency.

Warring et al. [11] tested the effectiveness of comprehensive multicultural training on prospective educators and school psychologists. The course objectives included: understanding the customs of different cultural groups, recognizing prejudices and acts of discrimination, respecting diversity, and developing inclusive behaviors. Students enrolled in the course were given a pretest and a posttest to measure possible changes in multiculturalism. Specifically, this study focused on changes in awareness of personal attitudes towards racial and ethnic minorities, knowledge about racial and ethnic minorities, and developing skills to effectively work with racial and ethnic minorities. A significant increase in levels of awareness, knowledge, and skill was observed amongst participants, which support the incorporation of multicultural courses in the school curriculum.

Multiculturalism interventions have benefits aside from increased levels of multicultural awareness. A study conducted by Turner and Brown [12] evaluated the effects of an intervention designed to improve elementary students’ attitudes toward refugees. The program, entitled the Friendship Project, aimed to teach students about the culture, lifestyle, and experiences of refugees [12]. Important dimensions of the program were developing knowledge about refugees, encouraging respect and empathy towards refugees, and gaining skills to detect biases and prejudices. Students were required to complete attitude measures before and after the implementation of the program. Participation in the Friendship Project resulted in positive, short-term attitudes towards refugees. The findings of this study demonstrate the beneficial effects of multiculturalism on attitudes and perceptions.

An experiment conducted by Hayes et al. [13] demonstrated the impact of multiculturalism on counselor burnout and stigmatizing attitudes towards substance abusers. The subjects were randomly assigned to one of the following conditions: Acceptance and Commitment Training (ACT), Multicultural Training, or Educational Control. Individuals in the multicultural condition were exposed to multiculturalism ideology, emphasizing cultural diversity, cultural competence, and awareness of personal values and biases. Assessments were completed before the training, after the training, and at a 3-month follow-up. The Multicultural Training condition was found to have a positive impact on stigmatizing processes [13].

Carter et al. [14] demonstrated that even a brief multiculturalism intervention could be beneficial in medical care. The program utilized in the study, The Cultural Proficiency Workshop, was a three-hour workshop implemented during a family medicine clerkship. The workshop focused on health disparities, enhancing cross-cultural awareness, and developing cross-cultural communication skills. Participants’ cultural attitudes and beliefs were assessed pre- and post-workshop, revealing an overall positive effect on participants’ cultural attitudes. Results indicated a positive change in self-awareness of cultural bias, as well as a greater awareness of culture’s role in medical care.

The effects of an interactive multicultural intervention on international students were examined by Sakurai, McCall-Wolf, and Kashima [15]. After the implementation of the multicultural program, participants developed more friendships and strengthened their orientation toward the local culture [15]; whereas non-participants showed a decrease in both of these factors and developed stronger home cultural orientation. The findings of this study suggest that similar interventions can be used to enhance social engagement among international students and can also be beneficial in building intercultural links between international and local students [15]. Overall, the studies above imply that teaching multiculturalism is an efficient way of addressing and combatting culturally based problems. The current study extended these previous intervention studies to develop an up-to-date, low-cost, and easy-to-administer virtual intervention specifically targeting college students.

### 1.3. Multiculturalism Components

The components of attaining multiculturalism are similar across cultural competency and multiculturalism literature. They emphasize acknowledging biases, inclusive behaviors, and cultural awareness. The common goal is to develop the necessary multicultural awareness, knowledge, and skills to respectfully engage with other cultural groups. This study will specifically utilize Carroll’s [16] multicultural flashpoints for change to develop a brief virtual multicultural training for college students. According to Carroll [16], the necessary skillsets for achieving multiculturalism include awareness, acknowledgment and knowledge, advocacy, and action.

Awareness, the first step towards multiculturalism, is pertinent in communication and developing respect for other cultures [16]. This stage involves awareness of personal values and recognizing oneself as a cultural being but is not limited to the awareness of self and personal beliefs. It also includes the awareness of others and their multiple cultural identities, systemic cultural bias issues, and future implications of relational cultural identities. In other words, learners in multicultural training must be aware of the existence of various cultures.

Another dimension is the acknowledgment and knowledge dimension. It is a two-stage cognitive process, which is ultimately affected by personal worldviews and one’s cultural self-awareness [16]. Acknowledgment involves the realization and acceptance of important cognitions regarding multiculturalism. It also involves the acquisition and reconsideration of knowledge. Acknowledging where one lacks in multicultural competence and seeking to gain more knowledge on the issue would be an example of applying this component.

The next stage towards transformational change in multiculturalism is advocacy. Carroll [16] described advocacy as “a process that takes one’s awareness, beliefs, knowledge, and acknowledgment and transforms them into a plan for effecting change” (p. 10). An example of advocacy relevant to our college intervention study would be promoting individuals’ advocacy actions in daily activities, such as standing up for a minority student who was teased because of their cultural background or promoting policy change in the university.

Taking action is the final multicultural flashpoint for change. This is the point where one becomes proactive in practicing multiculturalism. Carroll [16] considered action to be a broader set of events when compared to advocacy. It involves “a conscious, intentional, and deliberate act or activity” (p. 11) with widespread outcomes that benefit the lives of many and not just the individual. Although Carroll’s [16] flashpoints are specific to school professionals and personnel, our study aims at extending their application to university students. As detailed in Section 2.1, these flashpoints will serve as the foundation of the multiculturalism intervention in this study.

### 1.4. Study Design and Hypotheses

This study developed a virtual multiculturalism intervention and assessed how such intervention might improve individuals’ overall multicultural competence. The ultimate goal of this study was to have a positive effect on multiculturalism in university students and provide evidence of the benefits of incorporating multiculturalism in higher education. To achieve this, an intervention was developed and tested to assess if a virtual, multicultural-based task affects university students’ levels of multiculturalism. It was expected that in comparison to the control group, students’ multiculturalism score in the intervention group would increase after the intervention (Hypothesis 1).

In addition, the effectiveness of the multiculturalism intervention was also examined across race/ethnicities, genders, and years in college. Levels of multiculturalism have been shown to differ among races/ethnicities. For example, Pope-Davis et al. [17] examined multicultural counseling competence in Psychology students. Results indicated that ethnic minorities demonstrate higher levels of self-perceived multicultural competence in comparison to Caucasians. This study suggested potential racial differences in levels of multiculturalism in the present study. Another experiment conducted by Chao [18] supported the influence of race on multiculturalism. Specifically, a significant interaction between multicultural training and race/ethnicity was found to predict individuals’ levels of multicultural awareness. Therefore, it was expected that race would moderate the intervention’s effect on multiculturalism (Hypothesis 2). Specifically, it was predicted that racial minorities would have higher pre-intervention scores of multiculturalism compared to Caucasians, potentially creating a ceiling effect. Consequently, the intervention effect might be observed in Caucasians, instead of the racial minority sample.

In addition to race/ethnicity, gender is important to consider when examining multicultural competence. Nieto and Zoller Booth [19] analyzed the effects of cultural competence on international students. A significant difference in intercultural sensitivity among genders was observed—with females scoring higher on the sensitivity scale than males. This suggests the possibility of gender differences in multiculturalism in the current study. It was predicted that females would have higher multiculturalism levels pre-intervention compared to males. However, due to the possibility of a ceiling effect in females, the intervention may produce a more measurable change in males and not females (Hypothesis 3).

Similarly, it was expected that college experiences would moderate the intervention’s effect on levels of multiculturalism (Hypothesis 4). It was expected that the longer a student has been in college, the more exposure they would have to the university’s diversity office and other culture-related information. Therefore, higher pre-intervention multiculturalism scores were expected to be observed in students who have been in college longer. Due to the possibility of a ceiling effect in upper-class students, the intervention effect might be observed in students with less college experience. 

In summary, the current study attempted to examine the following research questions:

1. Do college students in the virtual multiculturalism intervention condition show greater improvement in multicultural competence, compared to college students in the control group? (Hypothesis 1)
2. Do race, gender, and years spent in college moderate the impact of the virtual multiculturalism intervention on college students’ multicultural competence? (Hypothesis 2–4)

## 2. Materials and Methods 

The Institutional Review Board (IRB) of the University of Louisiana at Lafayette approved the research under the approval number FA19-16 PSYC, “The effects of multicultural competence in university students”. Both the pilot study and the main study were approved under the same approval number.

### 2.1. Intervention Materials and Pilot Study

The virtual intervention was designed using Carroll’s [16] multicultural flashpoints for change: awareness, acknowledgment and knowledge, advocacy, and taking action. It was administered using Qualtrics. The intervention materials consisted of five major sets of activities, which were as follows: a diversity awareness quiz, “My Multicultural Self”, cultural sensitivity scenarios, educational videos on microaggressions, cultural appropriation, and implicit bias, and scenarios demonstrating advocacy and taking action. 

A pilot study was conducted to establish validity for the intervention materials. A total of 50 students who were comparable to the target sample of the main study (i.e., college students who are 18–24 years old) were recruited through the Psychology Department SONA participant pool. Using a 5-point Likert scale (strongly disagree to strongly agree), they were asked to rate each intervention material based on the material’s comprehensibility and overall effectiveness at meeting its main objective. To be included in the final intervention, an average rating of 4 or above in every aspect was required. The final intervention materials are as follows.

#### 2.1.1. Diversity Awareness Quiz

In this intervention, awareness was taught by utilizing the diversity awareness quiz, which was administered to facilitate awareness of systemic issues. The diversity awareness quiz [20] was intended to make participants aware of their lack of multicultural competence.

#### 2.1.2. My Multicultural Self

Awareness was also taught using an activity called “My Multicultural Self” [21]. This activity was administered to facilitate awareness of one’s own culture, of others’ multiple cultural identities, and stereotypes associated with cultural identities. In the pilot study, participants were presented with the “My Multicultural Self” activity.

#### 2.1.3. Cultural Sensitivity Scenarios

Awareness was also taught utilizing a cultural sensitivity scenario. This scenario aimed to enhance one’s cultural sensitivity and increase awareness of how individuals and their cultures can be misunderstood [22].

#### 2.1.4. Multiculturalism Educational Videos

The objective of the acknowledgment and knowledge dimension was for participants to acknowledge their lack of cultural competency and increase their knowledge of systemic cultural issues. Three cultural issues were covered in the intervention, including microaggressions, implicit biases, and cultural appropriation. Videos were approximately 3–6 min long.

#### 2.1.5. Advocacy and Taking Actions

Advocacy and action are closely related concepts, as taking actions involves putting advocacy into practice [16]. Therefore, they were combined in our intervention and were taught by providing scenarios illustrating microaggressions, implicit biases, and cultural appropriation. Participants were asked to choose the best way to respond to each scenario. Scenarios came from Oswald’s [23] Situational Judgment Inventory (SJI) measures and Carroll’s [16] chapter on multiculturalism.

### 2.2. Sample

A total of 350 undergraduate students were recruited. Only students who were 18 to 24 years old were included in the final sample. After excluding participants who spent less than 900 s (15 min) on the task, 249 participants were included in the final sample. Participants were randomly assigned to two groups (experimental or control). A total of 172 participants was assigned to the experimental condition (69%), and 77 participants were assigned to the control condition (31%). The average age of participants was 19.01 years (SD = 1.41). Out of the final sample, 148 participants (60%) identified as female, 97 participants identified as male (40%), and 1 participant identified as non-binary (0%). Participant race/ethnicity included Caucasian (*n* = 148, 60.0%), Black (*n* = 74, 30.0%), Hispanic (*n* = 10, 4.0%), Asian (*n* = 7, 28%), Native Americans (*n* = 2, 0.8%), and Multi-ethnic (*n* = 6, 2.4%). For analytic purposes, Black, Hispanic, Asian, and Multi-ethnic participants were categorized into one group (i.e., the racial minority group). In addition, 169 subjects were classified as freshmen (68%), 43 were sophomores (17%), 15 were juniors (6%), and 20 were seniors (8%). Most participants were non-immigrants (98%).

### 2.3. Procedure

Prior to the study, students were randomly assigned to either the experimental or control group. Random assignment was executed by participants drawing a slip of paper. The slips of paper were numbered from 1 to 20. Participants who drew a slip of paper with an even number were assigned to the experimental condition. Those who drew an odd number were assigned to the control condition. Then, students were presented with the consent forms.

Students’ multicultural competence was assessed before the virtual intervention or the control activity (i.e., pre-test). After the pre-test was completed, students in the experimental condition were directed to complete the virtual multiculturalism intervention. Participants in the control condition instead completed a 30 min survey that was unrelated to multiculturalism. After the intervention or the control activities, students’ multicultural competence was measured again (i.e., post-test). After the study, the participants were debriefed.

### 2.4. Measurements

The students’ level of multiculturalism was assessed pre- and post-intervention with the 18-item Munroe Multicultural Attitude Scale-Questionnaire (MASQUE) [24]. The MASQUE was tested for internal consistency and was found to be reliable (Cronbach’s Alpha = 0.72). Participants were asked to respond to these statements on a 6-point Likert-type scale, ranging from 1 (strongly disagree) to 6 (strongly agree). Sample items were “I realize that racism exists” and “I am emotionally concerned about racial inequality”. These responses were averaged to obtain a mean multiculturalism score for each participant. 

Gender, race/ethnicities, immigrant status, years in college, and age were included in the pre-test (i.e., before the multicultural intervention). The demographic information was used to examine whether there were significant group differences in multiculturalism improvement after the intervention.

### 2.5. Data Analysis

Repeated measures ANOVA was used to test the hypotheses. For testing Hypotheses 1 and 2 (conditions: intervention vs. no intervention) × 2 (time: pre- vs. post-test), a two-way repeated measures ANOVA was used to assess the difference in student multicultural competence before and after a virtual multiculturalism intervention in comparison to a control group. A significant interaction effect would indicate a significant intervention effect, and further pairwise comparisons would be conducted to unfold the pattern of the interaction. For testing Hypotheses 2 to 4, three-way repeated measure ANOVAs were conducted on race, gender, and years in college to determine the effect of each on the impact of the intervention (vs. control) on multicultural competence scores.

## 3. Results

### 3.1. Results of Hypothesis 1

**Figure 1:**
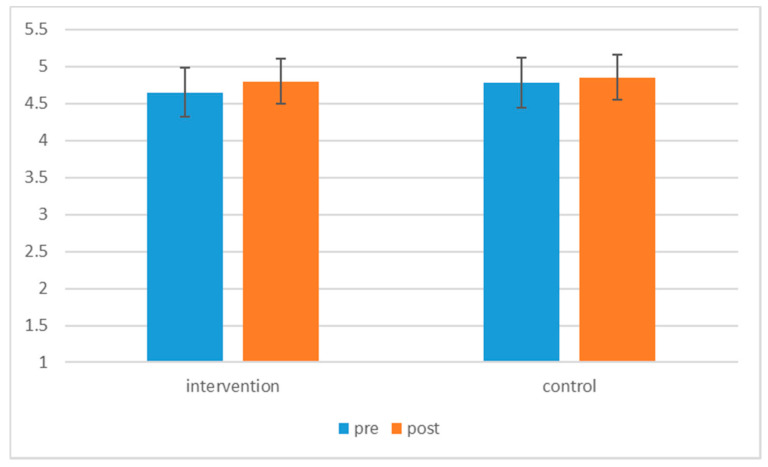
Significant intervention effect (pre- and post- overall multiculturalism scores by conditions).

A 2 × 2 repeated measures ANOVA was conducted using JMP Pro 14 to assess differences in multicultural competence between the experimental and control conditions across time, as shown in Table 1. The interaction effect of time by condition was found to be significant, F (1, 244) = 0.02, *p* = 0.048. Specifically, for those in the intervention condition, overall post-test scores (M = 4.80, SD = 0.67) were significantly higher than overall pre-test scores (M = 4.65, SD = 0.61), F (1, 169) = 26.06, *p* < 0.001. However, for those in the control condition, pre-test (M = 4.78, SD = 0.57) and post-test (M = 4.85, SD = 0.67) scores were not significantly different F (1, 75) = 2.43, *p* = 0.123. Results of the significant intervention effect are presented in Figure 1, and the means and standard deviations are presented in Table 2. For the main effects, the main effect of time, F (1, 244) = 0.07, *p* < 0.001, was significant, but the main effect condition was not significant, F (1, 244) = 0.001, *p* = 0.251. In other words, participants’ average multiculturalism scores in the intervention condition were not different from the scores of participants in the control condition. The results are in support of Hypothesis 1, which expected participants in the experimental condition to have a significant difference in multicultural competence scores pre- and post-intervention.

### 3.2. Results of Hypothesis 2

A three-way repeated measures ANOVA was conducted using JMP Pro 14 to assess the moderating effect of race/ethnicity on the intervention effect. The interaction of condition by race, F (1, 241) = 0.00, *p* = 0.507, and the interaction of all three factors (time, condition, and race), F (1, 241) = 0.04, *p* = 0.837 was also found to be non-significant. Therefore, race/ethnicity did not moderate the effect of condition or time or condition across time on the overall score of multiculturalism. The results did not support Hypothesis 2 of the current study, which expected racial minorities to have higher pre-intervention scores of multiculturalism compared to Caucasian participants. Results also showed that neither the main effects of condition, F (1, 241) = 0.00, *p* = 0.301, or the main effects of race, F (1, 241) = 0.02, *p* = 0.055 were significant.

### 3.3. Results of Hypothesis 3

A three-way repeated measures ANOVA was conducted to assess the moderating effect of gender (female vs. male) on the intervention effect. The interaction of all three factors (time, condition, and gender) was also found to be non-significant, F (1, 239) = 0.21, *p* = 0.651. Therefore, contrary to Hypothesis 3, gender did not moderate the effect of condition or time, or condition across time on the overall score of multiculturalism. Results also revealed that the main effect of condition F (1, 239) = 2.22, *p* = 0.138, and the interaction effect of condition by gender, F (1, 239) = 3.64, *p* = 0.058 were both non-significant. However, the main effect of gender was found to be significant, F (1, 239) = 16.55, *p* < 0.001. Specifically, females (Mpre = 4.84, SD = 0.57; Mpost = 4.97, SD = 0.63) scored higher than males (Mpre = 4.46, SD = 0.58; Mpost = 4.58, SD = 0.65).

### 3.4. Results of Hypothesis 4

A three-way repeated measures ANOVA was conducted using JMP to assess the moderating effect of college experience (upperclassmen vs. lowerclassmen). The interaction of all three factors (time, condition, and college experience) was found to be non-significant, F (1, 241) = 0.18, *p* = 0.675. Therefore, college experience did not moderate the effect of condition or time, or condition across time on the overall score of multiculturalism. Results also revealed the main effect of condition, F (1, 241) = 1.42, *p* = 0.235, and the interaction effect of condition by college experience to be non-significant, F (1, 241) = 0.27, *p* = 0.603. However, the main effect of college experience was found to be significant, F (1, 241) = 11.21, *p* = 0.001. Specifically, upperclassmen (Mpre = 5.00, SD = 0.56; Mpost = 5.15, SD = 0.68) scored higher than lowerclassmen (Mpre = 4.64, SD = 0.60; Mpost = 4.76, SD = 0.65).

## 4. Discussion

The present study aimed at developing a brief, online multiculturalism intervention for college students. The ultimate goal of this study was to have a positive effect on levels of multiculturalism in university students. It was hypothesized that in comparison to the control group, students’ multiculturalism score in the intervention group would increase after the intervention. The main significant findings of the study support the original hypothesis—with the intervention effect being observed only in participants in the intervention condition. These findings are consistent with previous intervention studies that have demonstrated the effectiveness of multiculturalism (i.e., [12,13,14]). 

In addition to the main hypothesis (Hypothesis 1), contradictory to Hypotheses 2–4, no moderating effects were found for race/ethnicity, gender, or college experience. It was hypothesized that the intervention would produce a more measurable change in certain groups of individuals (i.e., lowerclassmen, males, and Caucasians). However, the null findings of the current study suggest that the intervention is generally effective for all college students tested.

Although none of the hypothesized moderators were significant, some interesting significant main effects were observed. For example, the main effect of race was found to be marginally significant (*p* = 0.055). Specifically, racial minority participants scored higher than their Caucasian counterparts. Similar effects were observed with the main effect of gender and college classes. Specifically, females scored significantly higher than males on average multiculturalism scores. Finally, in comparison to those with less college experience (lowerclassmen), those with more college experience (upperclassmen) scored higher in the average scores of multiculturalism. Therefore, it appears that college education, in general, may help college students to gain knowledge in multiculturalism.

Although the main significant finding was relatively small, the current intervention is still beneficial to the college population, as it has shown to produce a measurable change in levels of multiculturalism. This intervention is very timely and useful to college students, as the US is in the midst of a global pandemic (i.e., COVID-19). Many occupational and educational tasks are now being implemented virtually. Being that this low-cost multiculturalism intervention is administered online, it can be easily adopted and accessed by individual students and universities alike.

Along with the COVID-19 pandemic, more awareness is being brought to the “Black Lives Matter” movement due to the increase in exposure to police brutality against Black American citizens. The mission of the “Black Lives Matter” movement is to bring awareness to anti-Black racism and state-sanctioned violence against Black Americans [25]. This movement exhibits a need for awareness of cultural differences in the US. The current multiculturalism intervention could be beneficial in increasing the awareness and acceptance of cultural differences. This could be a very useful tool for individuals and organizations in search of a cost-efficient tool to increase multiculturalism and raise awareness of systemic injustices without the need to have in-person diverse experiences.

### Limitations and Future Directions

It should be noted that the present study is limited in some respects. The study’s generalizability is limited, as most study participants were Caucasians and African Americans, and over half identified as female. The observed gender identity and ethnicity disparities do not provide an accurate representation of the diverse US population and could have possibly been resolved if more participants were recruited. Future studies should take sample size and its possible effects to external validity into consideration when developing a virtual intervention.

The current study could also be expanded longitudinally to gain a better understanding of assessing multiculturalism in college students. Instead of administering the intervention and assessing participants within the same day, the assessment and intervention processes could be carried out across longer periods (i.e., days or weeks). Giving participants more time to complete the intervention and delaying the pre- and post-assessments could result in higher attentiveness to the material presented. It can be expected that this would ultimately provide a more accurate representation of the intervention’s effects on multiculturalism.

Another possible limitation of the present study is related to the intervention being administered using an online-only platform. Prior research has demonstrated the effectiveness of in-person interactions on intergroup relations [26]. Specifically, properly structured contact between members of different cultural groups has been shown to improve intergroup attitudes, as well as reduce bias and conflict [26,27]. Therefore, the present intervention may not have been as effective as an intervention involving personal interactions. Future virtual multiculturalism intervention studies could incorporate activities that facilitate imagined intergroup contact, which has also been shown to be an effective strategy for improving intergroup relations [28]. Incorporating imagined contact activities would be pragmatic for a virtual intervention because it is timesaving and is as effective as direct intergroup contact.

## 5. Conclusions

This study attempted to develop a virtual multiculturalism intervention that increased levels of multiculturalism in college students. Ultimately, the intervention had a small significant effect on overall multiculturalism—with an increase in multiculturalism scores in only the experimental condition, yielding results that fall in line with this study’s hypothesis. This shows that there is a possibility of educating about multiculturalism within a university setting. Future interventions should also be conducted across a longer period of time by allowing more time for either the intervention or the assessment. Activities that involve intergroup contact (e.g., imagined intergroup contact) should also be incorporated to facilitate diverse experiences. This study contributes to the very limited literature on online multiculturalism interventions in undergraduate education and supports the incorporation of multiculturalism in higher education. It also demonstrates applicability to the current social and pandemic-related events occurring in the US.

## Figures and Tables

**Table 1:** The 2 × 2 repeated measure ANOVA results testing the intervention effect.

	*F*	*p*
Condition	0.01	0.251
Time	0.07	<0.001
Condition × Time	0.02	0.048

**Table 2:** Means and standard deviations of multiculturalism scores by time (pre- and post-), and F-statistics of the post-hoc analysis.

	Pre-Score	Post-Score		
	M (SD)	M (SD)	F	*p*
Intervention condition	4.65 (0.61)	4.80 (0.67)	0.07	<0.001
Control condition	4.78 (0.57)	4.85 (0.67)	0.01	0.251
